# Illustrations of the Elementary Forms of Disease

**Published:** 1834-04-01

**Authors:** 


*Illustrations of the Elementary Forms of Disease.* By Robert Carswell, m.d., Professor of Pathological Anatomy in the University of London, &c. Fasciculi I.—IV. London, 1833-4.

The fasciculi now before us contain an excellent account of Tubercles, Carcinoma, and Melanoma, illustrated by coloured plates, alike admirable for their truth and their beauty. The following observations on the curability of tubercular disease are remarkably interesting:

"Every physician must believe in the cure of scrofulous swellings, even without swelling or suppuration having taken place in them. Such cases, I am aware, are regarded by some as simple chronic inflammatory swellings of the lymphatic glands; but this opinion I by no means conceive to be correct, for, among the great number of cases which I have examined, I have never found these glands, when generally affected, exempt from the presence of tuberculous matter; and, even when the cutis is pale, (if they are situated under this tissue,) I have found them almost completely filled with this morbid product. When therefore enlarged glands in a scrofulous patient ultimately disappear, we may conclude, almost to a certainty, that we have witnessed the cure of a tubercular disease. Tabes mesenterica has been known to terminate favourably. I had an opportunity of examining, in a case of this kind, the mesenteric glands, and thereby of determining the certainty of the cure. The patient, who, when a child, was affected with this disease, and also swellings of the cervical glands, some of which ulcerated, died, at the age of twenty-one, of metritis, the seventh day after delivery. Several of the mesenteric glands contained a dry, cheesy matter, mixed with a chalky-looking substance; others were composed of a firm cretaceous substance; and a tumour, as large as a hen's egg, included within the folds of the peritoneum, and which appeared to be the remains of a large agglomerated mass of glands, was filled with a substance resembling a mixture of putty and dried mortar, moistened with a small quantity of turbid serosity. In the neck, and immediately behind an old cicatrix in the skin, there were two glands, which contained in several points of their substance (which was healthy,) small masses of hard cretaceous matter.

"I have also been able to trace the several steps of the same curative process in the bronchial glands, in individuals who had recovered from scrofula and pulmonary phthisis, but who died some time after of other diseases. I have found these glands situated at the bifurcation of the trachea, where they are generally most frequently and most extensively affected, as well as some way up the trachea, containing a greater or less quantity of a substance resembling putty or dry mortar, the consistence of which was sometimes equal to that of sandstone or bone. This substance has generally a stellated form, or presents a number of sharp spiculæ projecting from a central mass, which excite inflammation, ulceration, and hence perforation of the walls of the trachea or bronchial tubes with which they come in contact. A direct communication is thus formed between the cavity of the air-tubes and the diseased glands, through which the cretaceous bodies pass; and they are rejected along with the expectorated fluids. I have seen several examples of cure of tubercular disease of the bronchial glands effected in the manner just described. The patients were generally advanced in years, and had frequently observed the cretaceou matter in their sputa, portions of which have been shown to me, and were found to present all the physical characters of that which was afterwards detected in the bronchial glands.

"When these glands have evacuated the whole of their contents, they are found atrophiated, and converted into a fibrous tissue, which fills up the external orifice of the perforated air-tube. The accidental opening now contracts, becomes obliterated, and leaves in its place a puckered depression or cicatrix, seen on the internal surface of the air-tube.

"Similar appearances, indicating the removal of the serous and albuminous parts of the tuberculous matter, and the condensation of its earthy salts, have frequently been observed in the lungs of persons whose history left no doubt as to their having, at some period of their lives, been affected with tubercular phthisis. The important fact of the curability of this disease has, in my opinion, been already established by Laennec. I shall therefore only shortly allude to those changes which take place in the tuberculous matter, pulmonary tissue, and bronchi, which indicate that this fortunate termination of phthisis has taken place.

"The tuberculous matter, whether contained in the bronchial tube, the air-cells, or cellular tissue of the lungs, has assumed a dry, putty looking, chalky, or cretaceous character. If these changes are observed in an excavation, the surrounding pulmonary substance is generally dark-coloured; and, if the excavation exists in the course of large bronchial tubes, those situated between the excavation and the periphery of the lungs are obliterated, while those in the opposite direction terminate either in a short extremity near the excavation, or are continuous with the lining membrane or accidental tissue which encloses the altered tuberculous matter. The existence of this accidental tissue is an important circumstance as regards the cure of tubercular excavations. It is formed by the effusion of coagulable lymph on the surface of the excavation, or in the substance of the contiguous pulmonary tissue; has at first, and so long as a ready exit is afforded to its secretion, the characters of simple mucous tissue; but at a later period, and especially when the latter condition is wanting, it becomes gradually and successively converted into serous, fibrous, fibro-cartilaginous, and cartilaginous tissues.

"The cartilaginous and the osseous transformations of this accidental tissue are however rare, particularly the latter. It much more frequently retains the fibrous character, and possesses the property of contracting so as to diminish the bulk of the excavation, and carry with it the pulmonary tissue with which it is connected. The diminution of bulk which accompanies the removal of the tubercular matter, and the contraction of this accidental tissue, give rise to a puckering of the lungs, which is best seen where the pleura is forced to follow the retrocession of the pulmonary substance beneath it, and around what is called the cicatrix; for there sometimes remains only a small globular, oval, or even linear portion of fibrous or fibro-cartilaginous tissue, in a part of the lung where, from the extensive puckering around it, there must have formerly existed an excavation of considerable extent. When the tuberculous matter is contained within the bronchi, or a cavity formed by the dilatation of the air-cells, it does not appear that any accidental tissue is formed during the progress of the cure. The matter is gradually removed by expectoration, if the bronchi remain pervious, or by absorption, if they become closed; and then we have the same obliteration of the terminal branches already alluded to, and the same puckering of the surrounding tissues: all these appearances have been represented in plate iv., and will be better understood when pointed out in the figures."

The fasciculus on Melanoma, and the two which are devoted to Carcinoma, are also exceedingly good; but the long extract with which we have already gratified our readers forbids our indulging in any other one.

We trust that the Illustrations will meet with such patronage as may not only encourage Dr. Carswell to complete this great work, but may reward him for the immense labour which it must have cost him.

